# Plasma cytokine responses to resistance exercise with different nutrient availability on a concurrent exercise day in trained healthy males

**DOI:** 10.14814/phy2.13708

**Published:** 2018-06-05

**Authors:** Pim Knuiman, Maria T. E. Hopman, Roland Hangelbroek, Marco Mensink

**Affiliations:** ^1^ Division of Human Nutrition Wageningen University & Research Wageningen The Netherlands; ^2^ Department of Physiology Radboud University Medical Centre Nijmegen The Netherlands

**Keywords:** Circulating cytokines, endurance exercise, Nutrient availability, resistance exercise

## Abstract

Carbohydrate availability is proposed as a potential regulator of cytokine responses. We aimed to evaluate the effect of a preresistance exercise carbohydrate meal versus fat meal on plasma cytokine responses to resistance exercise after an endurance exercise earlier that day. Thirteen young, healthy, recreationally active males performed two experimental days with endurance exercise in the morning and resistance exercise in the afternoon. Either a carbohydrate (110 g carbohydrate, 52 g protein, 9 g fat; ~750 kcal) or an isocaloric fat meal (20 gr carbohydrate, 52 g protein, 51 g fat) was provided 2 h before resistance exercise. Blood was taken at baseline and at regular time intervals to measure circulating plasma cytokine levels (e.g. IL‐6, IL‐8, IL‐10, IL‐15, TNF*α*, ANGPTL4, decorin and MCP‐1). Plasma glucose and insulin were higher in the postprandial period before the start of the resistance exercise on the carbohydrate condition, while free fatty acids were reduced. At 2 h postresistance exercise, IL‐6 concentrations were higher in the fat condition compared to the carbohydrate condition (*P *<* *0.05). In addition, in both conditions IL‐6 levels were higher at all time points compared with baseline (*P *<* *0.05). The pattern of increase in plasma IL‐8 and IL‐10 did not differ significantly between conditions (*P *>* *0.05). There were no differences between conditions on TNF*α* levels and levels remain constant when compared with baseline (*P *>* *0.05). ANGPTL4, IL‐15, Decorin and MCP‐1 showed no differences between the fat and carbohydrate condition (*P *>* *0.05). The composition of the pre‐exercise meal did in general not influence cytokine responses in the postresistance exercise period, except postresistance exercise circulating plasma IL‐6 levels being higher in the fat condition compared with carbohydrate. Our findings support the view that pre‐exercise carbohydrate availability does not have a major impact on acute responses of circulating plasma cytokines in humans.

## Introduction

Cytokines are small proteins produced and released by several cells that play an integrative and regulatory role in local and systemic intercellular communication (Pedersen and Febbraio 2012; Hennigar et al. 2017). Up to now, many studies have assessed the effect of endurance and resistance exercise on circulating concentrations of cytokines (Peake et al. 2015). Even though the precise biological function of the majority of the cytokines is presently unclear, exercise‐induced elevations of circulating cytokine concentrations such as interleukin 6 (IL‐6), 8 (IL‐8), 15 (IL‐15), decorin, monocyte chemoattractant protein‐1 (MCP‐1) and 4 (MCP‐4) may play a role in adaptation of skeletal muscle (Catoire and Kersten 2015; Gorgens et al. 2015; Hennigar et al. 2017). However, the proposed role of cytokines in skeletal muscle regulation is mainly derived from mechanistic cell and animal studies, as a consequence, data from human studies is highly limited.

It is well established that exercise parameters such as mode, intensity, duration and type of exercise largely modulate cytokine response following exercise (Peake et al. 2015). In addition, nutrition (e.g., carbohydrates) appears to play a role as well in the modulation of circulating cytokines following exercise, particularly IL‐6 (Hennigar et al. 2017). For instance, circulating IL‐6 concentrations decrease with glucose ingestion during endurance exercise (Febbraio et al. 2003; Nieman et al. 2003), whereas low pre‐exercise muscle glycogen content augments these responses (Keller et al. 2001; Steensberg et al. 2001). Furthermore, work by Nieman et al. (2004) demonstrated that carbohydrate ingestion before and during a 2 h intensive resistance training did not alter circulating plasma levels of IL‐1ra, IL‐6, IL‐8, IL‐10 (Nieman et al. 2004). Remarkably, the role of pre‐exercise fat intake on plasma cytokines remains to be elucidated.

To our knowledge, not much work has been done on the effects of pre‐exercise nutrient availability on circulating cytokine concentrations following resistance exercise in humans. Since some of these cytokines appear to play a role in skeletal muscle growth, we were specifically interested in the effects of nutrient availability on circulating cytokines that have been implicated to play a role in muscle reconditioning. In our study, we aimed to evaluate the effects of a preresistance exercise carbohydrate meal versus fat meal on circulating cytokine concentrations to resistance exercise after an endurance exercise earlier that day. We designed an experimental protocol where endurance and resistance exercise were performed in the morning and afternoon, respectively, with ample protein ingestion throughout the day. The nutritional difference between the conditions was induced by a preresistance exercise meal that differed in macronutrient content. Specifically, in one condition the subjects were provided with a meal rich in carbohydrate (CHO condition), whereas in the other condition an isocaloric meal high in fat (FAT condition) was provided. It is hypothesized that some of the selected cytokines will differ between nutritional conditions, but that the majority of the circulating cytokines with a possible role in skeletal muscle growth do not respond differently.

## Methods

### Subjects

Fourteen healthy recreationally active males volunteered to participate in this study (age; 21.2 ± 0.5 years, height; 1.87 ± 0.0 m, mass; 76.7 ± 1.3 kg). One subject dropped out after test day 1 because of the discomfort of the muscle biopsies, therefore final analysis was performed on 13 subjects. All subjects were nonsmokers, free of injury and not taking any medication or nutritional supplements. All subjects provided full‐written informed consent. The Medical Ethical Committee of Wageningen University approved all study procedures.

### Subject characteristics

Physical characteristics of 13 volunteers are shown in Table**** 1.

**Table 1:** Physical characteristics of 13 volunteers

	Mean ± SE
Age (years)	21**.**2 ± 0.5
Height (m)	1**.**87 ± 0**.**0
Weight (kg)	76**.**7 ± 1.3
BMI (kg/m^2^)	22**.**0 ± 0.2
W_max_ (W)	346 ± 7.6
VO_2max_ (mL/kg/min)	51**.**3 ± 1.3
Leg press 1‐RM (kg)	266 ± 8.2
Leg extension 1‐RM (kg)	111 ± 3.0

### Study design

**Figure 1:**
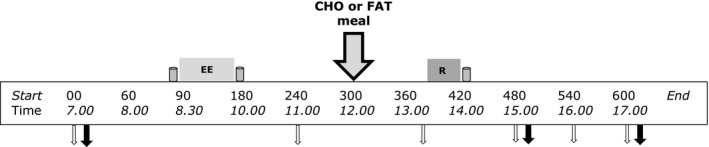
Schematic diagram of the experimental trials. This study adopted a counterbalanced crossover design where subjects completed both exercise trials with different nutritional treatments on separate occasions. Endurance exercise session: 90‐min cycling at 70% *V*O_2max_. Resistance exercise sessions 5 sets of 8 repetitions for both leg press and leg extensions at 80% 1‐RM. Gray containers indicates protein beverage (from left to right; 10 g casein, 15 g casein and 25 g whey. Big gray arrow with black outline indicates lunch with carbohydrates or fat (nutritional intervention meal). Open white arrows indicates time point for blood sample; black arrow indicates sampling time point for muscle biopsy.

This study used a randomized counterbalanced crossover design (Fig. 1). On both experimental days subjects completed the same exercise sessions: an endurance exercise session in the morning and a resistance exercise session in the afternoon with a resting period of 4 h between sessions. The subjects received a nutritional treatment in between the exercise sessions (carbohydrate or fat meal). Each trial was separated by a minimum of 12 days (range: 12–30 days), during which time the subjects were instructed to maintain their habitual lifestyle.

### Preliminary testing

#### Maximal aerobic capacity (*V*O_2max_)

Preliminary testing was performed in the week prior to the start of the first experimental day. Following a 30‐min rest, subjects performed a ramped *V*O_2max_ test on an electrically braked cycle ergometer (Lode Excalibur, Groningen, The Netherlands). After a 5‐min warm up at 50 W, the subjects started cycling at 100 W. Workload was progressively increased by 20 W/min until the subject reached volitional exhaustion. The *V*O_2max_ test was considered to be valid when two out of three criteria were met: I) levelling of *V*O_2_ with increasing workload; II) heart rate within 10 beats of the theoretically estimated maximum (220‐age) and III) respiratory exchange ratio (RER) of ≥1.15. Oxygen consumption (*V*O_2_) was measured through breath‐by‐breath sampling with an Oxycon Pro (Jaeger, Hoechberg, Germany) to define maximal oxygen consumption (*V*O_2max_). Subjects were asked to maintain a cadence between 80 and 100 r/min.

#### Maximal strength (1RM)

Maximal muscle strength was determined by one‐repetition maximum (1RM) strength tests on leg press and leg extension machine (Technogym, Cesena, Italy). Subjects were familiarized with the movement and warmed up prior to testing by performing six repetitions (at ~40% of estimated 1RM) through a full range of motion with a 1‐min rest. After each successful lift the weight was increased until a failed attempt occurred with 3‐min recovery between each attempt. The 1 RM was attained within five attempts. For each preliminary test, subjects were fasted for at least 4 h and were instructed to avoid strenuous physical activity 48 h before.

### Diet and physical activity control

Subjects were instructed to avoid alcohol, caffeine and physical activity during 48 h prior to the two main experimental trials. Diet and physical activity levels were recorded for 24 h before the first experimental trials and subjects were asked to replicate dietary intake and physical activity prior to the second experimental trial. Dietary intake records were analyzed, using Eetmeter Software 2005 (version 1.4.0;Voedingscentrum). A standardized meal for the subjects was provided the night before each trial (standard deep‐frozen meal and ice‐cream dessert; 43.80 kJ/kg BW; 15 Energy% protein, 30 Energy% fat and 55 Energy% carbohydrate).

### Experimental days

All subjects arrived at 07.00 in the morning in the fasted state at the laboratory of our university. After changing into sport gear, a catheter was inserted into the antecubital vein of the left arm. Fifteen minutes after insertion of the catheter baseline blood samples were taken followed by a baseline muscle biopsy. Approximately, 10 min after the baseline biopsy a protein beverage containing 10 gram of casein dissolved in 350 mL water was consumed (*t* = −05 min). Within five minutes after consumption of the protein beverage, the subjects started with 90 min cycling (*t* = 0 min) at submaximal intensity (70% *V*O_2max_). If subjects could not sustain the intensity of at least 60 RPM, resistance was decreased with 20 W, and the same intensity protocol was repeated during the second training. All subjects performed same exercise intensity of both experimental days. Immediately after the endurance exercise bout, a beverage containing 15 g of casein dissolved in 350 mL water was consumed (*t* = 90 min). A second blood sample (∼25 mL) was taken 1 h after the end of the endurance exercise bout (*t* = 150 min). Two hours after the endurance exercise bout (*t* = 210 min) subjects were randomly provided with either a carbohydrate rich low‐fat meal or low‐carbohydrate fat meal (see intervention meal for details). Ninety minutes after the meal (*t* = 300 min) a third blood sample (∼25 mL) was taken. At *t* = 330 min subjects performed the resistance exercise bout that consisted of 5 × 8 (80% 1RM) repetitions of bilateral leg press and leg extension with two minutes of rest in between the sets. If subjects could not sustain due to fatigue, the selected weight was decreased with steps of 10 kg or 5 kg for the leg press and leg extension, respectively. Immediately after the resistance exercise bout (*t* = 360 min), a protein beverage containing 25 g of whey protein dissolved in 350 mL water was consumed. One and three hours after the resistance exercise bout a second (*t* = 480 min) and a third (*t* = 600 min) skeletal muscle biopsy was taken. All biopsies were taken from the same leg. Additional blood samples were taken at *t* = 480, 540 and 600 min. A timeline for the experimental day can be found in Figure 1. The whole experimental protocol was repeated on the 2nd day, while the other meal was provided.

### Nutritional intervention

Both meals were cooked prepared by a research dietician of Wageningen University. Both the carbohydrate and fat meal consisted of commercially available meat, macaroni and vegetables with an energetic value of ∼3200 kJ. The absolute macronutrient amounts were chemically analyzed as described previously (Koreissi‐Dembele et al. 2017) and can be found in Table 2.

**Table 2:** Overview of the energy and macronutrient composition intervention meal

Energy & nutrient	Fat meal	Carbohydrate meal
Energy (kJ)	3207	3124
Protein (g)	52	52
Fat (g)	51	9
Carbohydrates (g)	20	110

### Exercise protocol

Since athletes commonly combine divergent exercise sessions on the same day, we decided to include both endurance and resistance exercise on the same day in our protocol. Additionally, it has been recently proposed that, when combining divergent exercise sessions within the same day, the endurance session should be performed in the morning in the fasted state, with ample protein ingestion, while the afternoon resistance exercise session should be conducted only after carbohydrate replenishment with adequate postresistance exercise protein ingestion (Perez‐Schindler et al. 2015). With our study we aimed to evaluate the effect of a preresistance exercise isocaloric (~3200 kJ) mixed meal containing different amounts of carbohydrates and fat, on postresistance exercise plasma cytokines after glycogen depleting exercise earlier that day.

### Muscle biopsies

Muscle biopsies were taken as described by Bergstrom (1975). Biopsies were taken under local anesthesia (2–3 mL of 2% Xylocaine) using a 5‐mm Bergstrom needle modified with suction. All three muscle biopsies on one experimental day were taken from the vastus lateralis of the same leg, with separate incisions (~1–1.5 cm) apart and from distal to proximal direction. Muscle biopsies on the second test day were taken from the contralateral leg. Muscle biopsies were immediately frozen (in 5–10 sec) in liquid nitrogen and stored at −80°C for subsequent biochemical analysis, after being freed from visible fat, blood, and connective tissue.

### Muscle glycogen

Muscle tissue, ~30 mg wet weight, was freeze dried, after which collagen, blood, and nonmuscle fiber materials were removed from the muscle fibers under a microscope. The isolated muscle fiber mass (~5–7 mg) was weighed, and 500 *μ*L of 1 MHCI was added. After heating for 3 h at 100°C to hydrolyze the glycogen to glycosyl units and cooling down to room temperature, 500 *μ*L of the solution was neutralized by adding 280 *μ*L of Tris–KOH (Tris 119 mmol/L, KOH 2.14 mol/L) and centrifuged at 1000 *g* and 4°C for 10 min. Thereafter, 150 *μ*L of this solution was analyzed for glucose concentration (Glucose HK CP A11A01667, ABX Pentra) with a COBAS FARA semiautomatic analyzer (Roche).

### Glucose, insulin and free fatty acids

Blood was collected in EDTA‐containing tubes. The samples were immediately centrifuged at 1000 g at 4°C for 10 min, after which plasma was stored in −80°C until further analysis. Blood samples were analyzed for glucose and insulin (Gelderse Vallei hospital, Ede, NL). Free fatty acids were assessed using an enzymatic test kit according to the manufacturer's protocol (InstruChemie, Delfzijl, Netherlands).

### ANGPTL4 and Decorin

All plasma samples were kept on ice until centrifugation and stored at −80°C until further analysis. Angiopoietin‐like 4 (ANGPTL4) and Decorin were assessed using human ELISA kits according to the manufacturer's protocol.

### IL‐6, IL‐8, IL‐10, IL‐15,MCP‐1, and TNF‐*α*

Blood samples were taken at baseline, 1 h postendurance exercise, 1 h postmeal, 1 h, 2 h and 3 h postresistance exercise. Participants were seated for 5 min after which blood samples were taken from the cephalic vein. Blood was collected in a 4 mL EDTA vacutainer (Becton‐Dickinson, New Jersey, America). The vacutainer was immediately put on melting ice water (4°C) and centrifuged at 1200*g* for 15 min at 4°C. Serum was transferred to polypropylene tubes and stored at −80°C until Cytokine analysis. We measured IL‐6, IL‐8, IL‐10, IL‐15, MCP‐1, and TNF‐*α* concentrations, using the ultrasensitive MesoScale Discovery (MSD) QuickPlex SQ 120 Instrument with Multispot assay (Human Proinflammatory Panel 1, K15049D, MSD) according to the manufacturers’ recommendations. The lower detection limit was 0.043–0.107, 0.039–0.059, 0.022–0.034, 0.46–0.54, 0.09–0.16 and 0.055–0.087 pg/mL, respectively, varying per plate. Precision of these validated kits was as follows: The intra‐run % CV for the high‐low controls were 3.6–4.5%, 2.7–3.0%, 2.6–3.7%, 3.3–4.1% and 2.7–3.4% for IL‐6, IL‐8, IL‐10, IL‐1*β*, and TNF‐*α*, respectively. The Inter‐run % CV for the high‐low controls were 5.2–7.3%, 5.0–7.1%, 5.7–10.1%, 5.5–7.7% and 6.1–10.1% for IL‐6, IL‐8, IL‐10, IL‐1*β* and TNF‐*α*, respectively. With the high‐low concentrations being as follows: 239–18.4 pg/mL, 166–12.5 pg/mL, 107–7.18 pg/mL, 152–11.2 pg/mL, and 75.5–4.45 pg/mL, for IL‐6, IL‐8, IL‐10, IL‐1*β*, and TNF‐*α*, respectively.

### Statistics

Data were analyzed using a two‐way repeated measures ANOVA (two factor, time x treatment) from IBM SPSS version 23 statistical software (IBM, Armonk, NY). When a main effect of trial of time or interaction was identified, a pairwise multiple comparisons with a Bonferroni correction was done to identify differences. Statistical significance was set at the *P* < 0.05 level, and values were expressed as means ± SEM or different when indicated.

## Results

### Endurance and resistance exercise performance

Two subjects performed the endurance exercise sessions with a reduced workload. For one subject the workload was reduced with 20 W after 20 min whereas for the other subject the workload was reduced with 20 W after 30 min. There was no further reduction in workload during the remaining part of the endurance session. Additionally, both subjects were able to repeat this on the second experimental day. All 13 subjects performed the resistance exercise training with exactly the same amount of work on the 2 experimental days.

### Muscle glycogen

**Figure 2:**
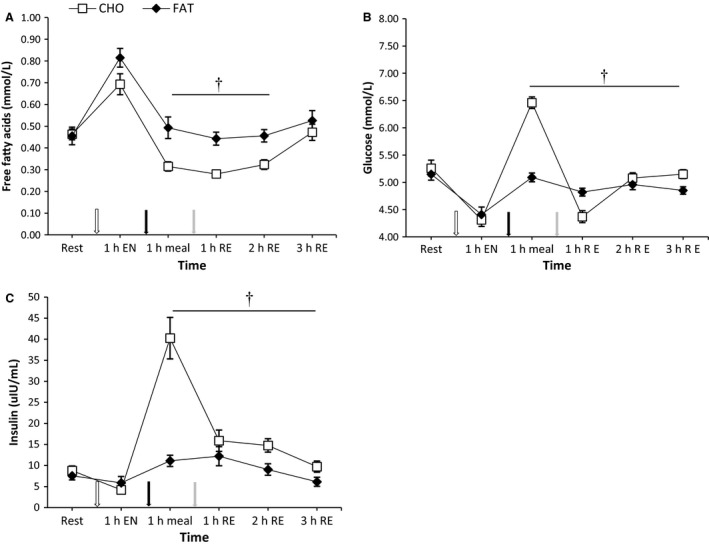
Mixed muscle glycogen at rest and 1 h and 3 h after 5 sets of 8 repetitions for both leg press and leg extensions at 80% 1RM (1, 2 and 3 h RE) Values are mean ± SEM. *Significantly different (*P* < 0.05) versus rest. †Significantly different (*P* < 0.05) between conditions (interaction). CHO = open bars, FAT = filled bars.

There were no differences in baseline muscle glycogen between the conditions (carbohydrate 380 ± 20.4 mmol/kg dry weight (dw) versus fat 441 ± 26.3 mmol/kg dw; *P *>* *0.05 Fig. 2). As a result of endurance exercise, muscle glycogen was significantly reduced compared to baseline in both conditions at 1 h and 3 h postresistance exercise (*P *<* *0.05) (carbohydrate 163 ± 23.5 and 181 ± 20.3 mmol/kg dw; fat 185 ± 25.8 and 140 ± 24.4 mmol/kg dw), without any significant differences between the conditions (*P *>* *0.05).

### Free fatty acids, glucose and insulin

**Figure 3:**
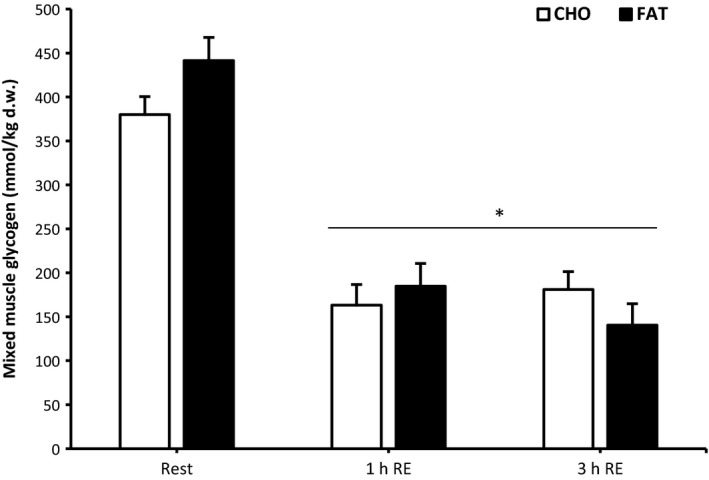
Plasma free fatty acids, glucose and insulin at rest and 1 h after 90‐min cycling 70% *V*O_2max_ (1 h EN), 1 h postmeal (1 h meal) and 1 h,2 h, and 3 h after 5 sets of 8 repetitions for both leg press and leg extensions at 80% 1RM (1,2 and 3 h RE) Values are mean ± SEM. *Significantly different (*P* < 0.05) versus rest. †Significantly different (*P* < 0.05) between conditions (interaction). White arrow: endurance exercise session; black arrow: nutritional intervention meal; gray arrow: resistance exercise session.

One hour postmeal, plasma free fatty acid concentration was higher in the fat condition compared with the carbohydrate condition and this effect remained significant after 1 and 2 h postresistance exercise (*P *<* *0.05) (Fig. 3). Both glucose and insulin in the fat condition were significantly lower at 1 h postmeal, and at 1, 2 and 3 h postresistance exercise compared with the carbohydrate condition (*P *<* *0.05) (Fig. 3).

### IL‐6, IL‐8, IL‐10 and TNF*α*

**Figure 4:**
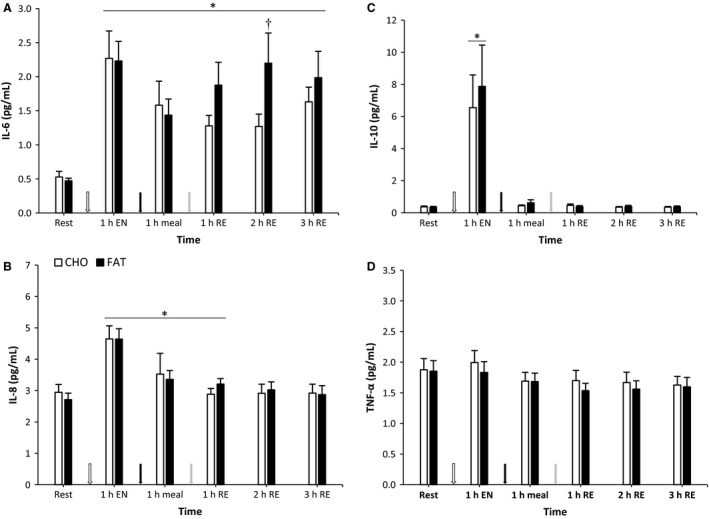
IL‐6, IL‐8, IL‐10 and TNF *α* at rest and 1 h after 90‐min cycling 70% *V*O_2max_ (1 h EN), 1 h postmeal (1 h meal) and 1 h,2 h, and 3 h after 5 sets of 8 repetitions for both leg press and leg extensions at 80% 1RM (1,2 and 3 h RE) Values are mean ± SEM. *Significantly different (*P* < 0.05) versus rest. †Significantly different (*P* < 0.05) between conditions (interaction). White arrow: endurance exercise session; black arrow: nutritional intervention meal; gray arrow: resistance exercise session.

At 2 h postresistance exercise, IL‐6 concentrations were higher in the fat condition compared to the carbohydrate condition (*P *<* *0.05) (Fig. 4). In addition, in both conditions IL‐6 levels were higher at all time points compared with baseline (*P *<* *0.05). The pattern of increase in plasma IL‐8 and IL‐10 did not differ significantly between conditions (*P *>* *0.05) (Fig. 4). As a result of the endurance session in the morning, concentrations of both IL‐8 and IL‐10 were increased 1 h postendurance exercise (*P *<* *0.05). Furthermore, compared to baseline, IL‐8 was still increased 1 h postmeal and 1 h postresistance exercise (*P *<* *0.05), with no differences between meal conditions (*P *>* *0.05). There were no differences between conditions on TNF*α* levels and levels remain constant when compared with baseline (*P *>* *0.05).

### ANGPTL4, IL‐15, Decorin and MCP‐1

**Figure 5:**
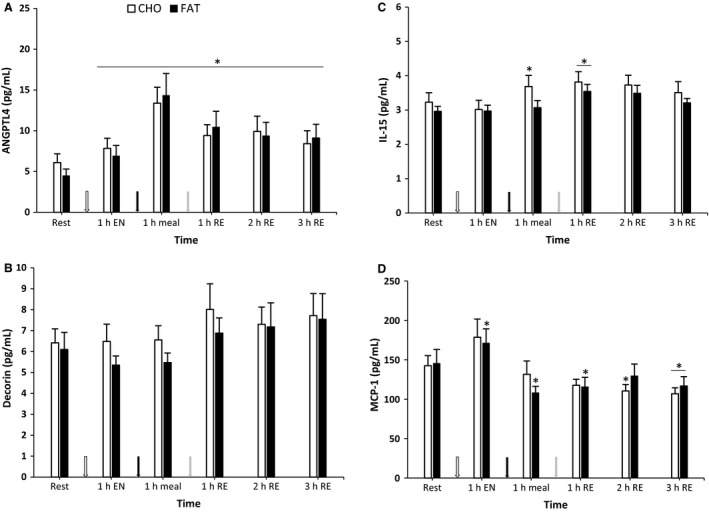
ANGPTL4, Decorin, MCP‐1 and IL‐15 at rest and 1 h after 90‐min cycling 70% *V*O_2max_ (1 h EN), 1 h postmeal (1 h meal) and 1 h,2 h, and 3 h after 5 sets of 8 repetitions for both leg press and leg extensions at 80% 1RM (1,2 and 3 h RE) Values are mean ± SEM. *Significantly different (*P* < 0.05) versus rest. †Significantly different (*P* < 0.05) between conditions (interaction). White arrow: endurance exercise session; black arrow: nutritional intervention meal; gray arrow: resistance exercise session.

ANGPTL4, IL‐15, Decorin and MCP‐1 showed no differences between the fat and carbohydrate condition (*P *>* *0.05) (Fig. 5). ANGPTL 4 levels were higher compared to baseline in both conditions at all time points (*P *<* *0.05). IL‐15 was higher than baseline in the carbohydrate condition 1 h postmeal and 1 h postresistance exercise, whereas in the fat condition an increase compared with baseline was found only 1 h postresistance exercise (*P *<* *0.05). Decorin levels were not different from baseline at any time point for both conditions (*P *>* *0.05). MCP‐1 levels were higher than baseline in the fat condition at any time point except 2 h postresistance exercise (*P *<* *0.05). In the carbohydrate condition at 2 and 3 h postresistance exercise the MCP‐1 levels were elevated compared to baseline (*P *<* *0.05).

## Discussion

In this study, we investigated whether pre‐exercise nutrient availability such as carbohydrates and fat influenced circulating cytokines concentrations with resistance exercise on a concurrent exercise day. We observed that the majority of the circulating cytokines (ANGPTL4, decorin, IL‐8, IL‐10, IL‐15, MCP‐1 and TNF‐*α*) are not affected by differences in preresistance exercise carbohydrate or fat availability during the early postresistance exercise recovery period on a concurrent exercise day. We were especially interested in circulating IL‐6, IL‐8, IL‐15, decorin and MCP‐1, since elevations of these cytokines may contribute or reflect adaptations of skeletal muscle (Catoire and Kersten 2015; Perez‐Lopez et al. 2017). The only cytokine of our selective set of circulating cytokines that responded differently between the nutritional conditions was IL‐6.

### Cytokines that have been implicated in skeletal muscle hypertrophy

IL‐6 is one of the most discussed cytokines (Hennigar et al. 2017) that responds primarily to acute muscle contraction and peaks immediately after exercise (Pedersen and Febbraio 2012), as was also observed in our study after the endurance session in the morning. In addition, it can be assumed that the significant reduction of hepatic and skeletal muscle glycogen levels postendurance exercise contributed to the observed rise in plasma IL‐6. Previous animal‐ and cell‐culture work showed that IL‐6 is probably an important regulator of satellite cell‐mediated skeletal muscle hypertrophy (Serrano et al. 2008). We found that two hours postresistance exercise circulating IL‐6 was significantly higher in the low carbohydrate condition compared with the high carbohydrate condition. Although we cannot exclude the possibility that this was partly due to a difference in muscle glycogen content during the resistance exercise bout, it is most likely due to the difference in carbohydrate availability in the pre‐exercise meal since we found no differences in skeletal muscle glycogen in the postresistance exercise period. Indeed, increased circulating levels of IL‐6 have been found to be sensitive to changes in blood glucose levels (Febbraio et al. 2003), but also to pre‐exercise muscle glycogen content (Steensberg et al. 2001). Likewise, pre‐exercise reduced intramuscular glycogen augments IL‐6 mRNA transcription factors and plasma IL‐6 concentrations (Keller et al. 2001; Chan et al. 2004), possibly explained through activation of AMPK (MacDonald et al. 2003) and p38MAPK (Chan et al. 2004). In addition, increased plasma IL‐6 may act as a signal for hepatic glucose release to maintain blood glucose levels during exercise (Hennigar et al. 2017). Since we compared two isocaloric meals with different amounts of carbohydrates we cannot conclude whether the low carbohydrate availability augmented expressions of IL‐6 plasma or that the high carbohydrate availability attenuated IL‐6 plasma levels.

In addition to circulating IL‐6 we had particular interest in the responses of circulating IL‐15 since this cytokine has been implicated in the regulation of skeletal muscle turnover (Quinn et al. 1995, 2002; Furmanczyk and Quinn 2003; Riechman et al. 2004; Busquets et al. 2005; Pistilli et al. 2007). The responses of the circulating IL‐15 with exercise vary between studies, but it seems that IL‐15 primarily responds to resistance exercise in both trained and untrained individuals (Riechman et al. 2004; Nielsen et al. 2007). Overexpression of IL‐15 in cultured skeletal myotubes affects protein metabolism by stimulation of protein synthesis and inhibition of protein degradation, suggesting a possible role for IL‐15 in muscle growth and wasting (Quinn et al. 2002). Furthermore, it has been suggested that IL‐15 plays a role in muscle adipose tissue interaction (Argiles et al. 2005). Our plasma data of IL‐15 showed an increase at 1 h postresistance exercise without a difference between the carbohydrate and fat condition suggesting that circulating plasma IL‐15 is not affected by the preresistance exercise difference in availability of carbohydrate or fat. The evidence that IL‐15 plays an important role in skeletal muscle growth with exercise is mainly derived from in vitro and animal work and its relevance in terms of physiological adaptation in humans remains to be determined (Bell et al. 2000; Quinn et al. 2002; Furmanczyk and Quinn 2003; Pistilli and Alway 2008).

The majority of the selected cytokines responded as a result of exercise, this was also the case for circulating decorin. We found a mild increase, although not significant in plasma decorin at 1, 2 and 3 h postresistance exercise when compared to circulating levels of decorin at baseline. However, no differences were found between the low and high carbohydrate condition. Decorin is a protein secreted by skeletal muscle cells, and promotes skeletal muscle hypertrophy by binding with myostatin (Kanzleiter et al. 2014). It has recently been demonstrated that plasma decorin increases in response to acute resistance exercise (Kanzleiter et al. 2014). The somewhat moderate response in our study of plasma decorin compared to the study of Kanzleiter et al. (2014) could be explained by exercise volume and time points of measurement. Indeed, Kanzleiter et al. (2014) used seven different exercises of three sets and measured plasma decorin during and immediately after, every 30 min until 120 min postresistance exercise. In their study, which clearly had a higher exercise load, plasma decorin peaked immediately after exercise, a time point we are lacking in our study (Kanzleiter et al. 2014). The last cytokine we investigated with a possible role in skeletal muscle growth is MCP‐1. Previous studies show that MCP‐1 is both a cytokine and exercise factor. MCP‐1 appears to be vital for muscle recovery and adaptation (Staiger et al. 2009; Schwarz et al. 2010). Divergent exercise modes increase skeletal muscle gene expression levels of MCP‐1 (Suzuki et al. 2003; Tantiwong et al. 2010; Mathers et al. 2012; Vella et al. 2012). Yet, MCP‐1 responded as a result of exercise while no differences were seen between the nutritional conditions.

### ANGPTL4

In our nutritional intervention we compared two isocaloric pre‐exercise meals that differ in macronutrient profile. Specifically, in the FAT condition, the carbohydrates were almost completely replaced by fat. Therefore, we also looked to the responses of ANGPTL4 since this protein appears to be sensitive to glucose ingestion (Kersten et al. 2009). ANGPTL4 is found in both skeletal muscle and adipose tissue and is regulated by exercise via free elevated free fatty acids (Kersten et al. 2009). ANGPTL4 stimulates degradation of lipids and thereby the release of glycerol and free fatty acids to the circulation. Furthermore, circulating plasma ANGPTL4 responds to exercise and previous work by Kersten et al. (2009) demonstrated that exercise‐induced ANGPTL4 plasma responses are partly inhibited when subjects are given oral glucose (Kersten et al. 2009). It is theorized that glucose inhibits ANGPTL4 possibly caused by the rise of insulin, via suppression of lipolysis and reduced plasma free fatty acids concentration (Kersten et al. 2009; Catoire et al. 2014). Our data confirmed findings of others that plasma ANGPTL4 increases significantly after endurance exercise (Kersten et al. 2009). The increase in ANGPTL4 was further elevated 1 h postmeal possibly because of a positive feedback loop of plasma free fatty acids raising ANGPTL4 levels and ANGPTL4 promoting adipose tissue lipolysis, raising free fatty acids plasma levels (Kersten et al. 2009). Despite a significant difference in circulating free fatty acids, glucose and insulin between feeding conditions, no differences in plasma ANGPTL4 were detected.

### Study limitations

A limitation of the present study is that there was no muscle biopsy taken preresistance exercise and therefore the muscle glycogen content between the different nutritional conditions when commencing the resistance exercise bout remains unclear. Furthermore, our blood samples in the postexercise period were not immediately collected after the bout but at 1, 2 and 3 h postexercise. Consequently, potential differences in cytokines between the nutritional conditions could have been overlooked since the established cytokines reach a peak during and immediately after exercise (Pedersen and Febbraio 2012). In addition, the effects of the nutritional intervention are possibly obscured by the physiological effects of endurance exercise. Multiple time points in a shorter period of time during and after exercise may reveal more additional insights about how nutrient availability influence cytokine responses. Another limitation is the volume and intensity of the resistance exercise bout, we cannot rule out the possibility that alternative resistance exercise bouts higher in volume and intensity with different carbohydrate or fat availability may affect the postresistance exercise cytokine responses differently. Moreover, the postresistance exercise plasma cytokine responses may be affected by the endurance exercise earlier that day, however, since we did not have a third condition without the endurance exercise we were not able to correct for this potential confounder. At last, another shortcoming of this study is that possible changes within the muscle such as mRNA quantity and protein content were not determined. Therefore, a possible effect of the intervention meal may have been overlooked.

## Conclusion

In summary, resistance exercise with different carbohydrate/fat availability did not influence most plasma cytokine responses in the early postresistance exercise period. Postresistance exercise plasma IL‐6 expression was higher in the low carbohydrate condition compared with high carbohydrate availability. Our findings support the view that the preresistance exercise carbohydrate availability does not affect acute cytokine responses to resistance exercise.

## Conflict of Interest

The authors have no conflicts of interest to declare that are directly relevant to the contents of this manuscript.
